# Optimization of Magnetic Finishing Process and Surface Quality Research for Inner Wall of MP35N Cobalt–Chromium Alloy Vascular Stent Tubing Based on Plasma-Fused Al_2_O_3_ Magnetic Abrasives

**DOI:** 10.3390/mi16050591

**Published:** 2025-05-18

**Authors:** Yusheng Zhang, Yugang Zhao, Qilong Fan, Shimin Yang, Shuo Meng, Yu Tang, Guiguan Zhang, Haiyun Zhang

**Affiliations:** School of Mechanical Engineering, Shandong University of Technology, No. 266 Xincun West Road, Zibo 255049, China; zys19862734240@126.com (Y.Z.); fql0317@126.com (Q.F.); ysmsdut@126.com (S.Y.); m1369160775@126.com (S.M.); tangyu1232022@126.com (Y.T.); zhanggg1006@163.com (G.Z.); zhy@sdut.edu.cn (H.Z.)

**Keywords:** magnetic abrasive finishing, magnetic abrasive, MP35N cobalt–chromium alloy tube, surface roughness, response surface method

## Abstract

To solve the manufacturing problem of the efficient removal of multi-scale surface defects (wrinkles, cracks, scratches, etc.) on the inner wall of MP35N cobalt–chromium alloy vascular stents, this study proposes a collaborative optimization strategy of magnetic abrasive polishing (MAF) based on a new type of magnetic abrasive. In response to the unique requirements for the inner wall processing of high aspect ratio microtubes, metal-based Al_2_O_3_ magnetic abrasives with superior performance were prepared by the plasma melt powder spraying method. A special MAF system for the inner wall of the bracket was designed and constructed. The four-factor and three-level Box–Behnken response surface method was adopted to analyze the influences and interactions of tube rotational speed, magnetic pole feed rate, abrasive filling amount, and processing clearance on surface roughness (Ra). The significance order of each parameter for Ra is determined as follows: processing clearance > tube rotational speed > abrasive filling amount > magnetic pole feed rate. Using the established model and multiple regression equations, the optimal parameters were determined as follows: a tube rotational speed of 600 r/min, a magnetic pole feed rate of 150 mm/min, an abrasive filling amount of 0.50 g, and a processing clearance of 0.50 mm. The optimized model predicted an Ra value of 0.104 μm, while the average Ra value verified experimentally was 0.107 μm, with the minimum error being 2.9%. Compared with the initial Ra of 0.486 μm, directly measured by the ultra-depth-of-field 3D microscope of model DSX1000, the surface roughness was reduced by 77.98%. MAF effectively eliminates the surface defects and deteriorated layers on the inner wall of MP35N tubes, significantly improving the surface quality, which is of great significance for the subsequent preparation of high-quality vascular stents and their clinical applications.

## 1. Introduction

Cardiovascular diseases are the leading cause of death worldwide, with mortality rates increasing year by year in China. This rise is attributed to changes in lifestyle and an increase in the incidence of acute coronary syndromes [1,2,3]. For certain critically ill patients, surgical intervention and medical devices remain the only viable options, with stents becoming a primary method for treating cardiovascular diseases [4,5,6]. Currently, most metallic vascular stents on the market are manufactured from stent tubes created through laser cutting. These tubes are primarily formed by extruding and continuously drawing metal billets, which involves complex processing parameters. As a result, the inner walls of the formed tubes are prone to various surface defects, including pits, folds, cracks, scratches, and pitting, as well as the presence of a degraded layer of hard particle oxides. These defects hinder the subsequent processing of the vascular stents and complicate the materials’ post-treatment, thereby affecting their therapeutic efficacy in the human body.

Cobalt–chromium alloys are widely used in dental and orthopedic implants due to their superior mechanical properties, biocompatibility, and corrosion resistance [7,8,9]. Their high elastic modulus enables cobalt–chromium alloy vascular stents to be thinner and more flexible while maintaining high radial strength [9]. Furthermore, the high density of cobalt–chromium alloys enhances their compatibility with MRI and improves visibility under X-ray imaging. These characteristics make cobalt–chromium alloys an ideal choice for fabricating vascular stents, thereby enhancing patient safety and treatment outcomes. Consequently, eliminating surface defects on the inner walls of cobalt–chromium alloy tubes during the manufacturing process is crucial for achieving high-quality inner surfaces, particularly in the field of medical vascular stent fabrication.

Currently, electrochemical polishing is a commonly used method for polishing the inner walls of metal vascular stent tubes [10,11,12]. The principle involves using a vascular stent tube as the anode and applying a direct current in an electrolytic cell, which induces an oxidation reaction on its surface to remove defects and reduce surface roughness. However, this method has several issues, such as uneven surface oxide layers and the tendency to generate new defects, including impurities and blisters. As noted by Simka et al. [13], in their experimental study utilizing a concentrated sulfuric acid–alcohol system as the primary polishing solution, they compared the appearance of samples treated with three different electrochemical polishing solution formulations: concentrated sulfuric acid–nitric acid–hydrofluoric acid, concentrated sulfuric acid–hydrofluoric acid–methanol, and concentrated sulfuric acid–hydrofluoric acid–ethylene glycol. The study found that the concentrated sulfuric acid–nitric acid–hydrofluoric acid system exhibited excessive acidity, leading to uneven corrosion on the sample surface, resulting in significant and highly variable surface roughness. Babilas et al. [14] similarly proposed the use of this dual-acid system for sample polishing, achieving relatively uniform surfaces with good corrosion resistance. However, due to the strong corrosiveness of concentrated sulfuric acid and concentrated phosphoric acid, the surface condition of the alloy is susceptible to the effects of strong acid corrosion, leading to unstable polishing results. Improper control of polishing time may also result in over-polishing, while the presence of phosphorus (P) and sulfur (S) elements could cause environmental pollution. Furthermore, Fushimi et al. [12] discovered that adding a small amount of deionized water to a concentrated sulfuric acid and methanol solution while polishing at −10 °C could yield samples with higher surface gloss. However, methanol is highly toxic, and prolonged exposure to a methanol environment poses health risks. All these factors hinder subsequent coating and drug incorporation processes and may pose health hazards. Additionally, electrochemical polishing equipment is complex, requiring precise control of parameters such as the composition, concentration, and current density of the electrolyte, and involves the use of corrosive electrolytes that can cause environmental pollution. Due to equipment limitations, achieving efficient polishing for longer vascular stent tubes presents significant challenges. Therefore, for small-diameter seamless metal tubes, the search for more efficient, environmentally friendly, and safe methods and processes for inner wall finishing has become an important focus of current research.

Magnetic Abrasive Finishing (MAF) is an advanced processing technique that integrates the effects of magnetic fields with traditional grinding methods. This technology enables the enhancement of nanometer-level surface roughness with minimal damage to the workpiece surface. By harnessing the magnetic field’s influence, magnetic abrasives can penetrate non-magnetic materials like magnetic field lines. Specifically, when a magnetic field is applied externally to the workpiece, the internally filled magnetic abrasives become magnetized, forming numerous elastic magnetic abrasive brushes aligned along the magnetic field lines. These brushes press against the surface of the workpiece to be processed and move relative to it. During this interaction, the magnetic abrasives continually engage with the surface through processes such as pressing, scratching, sliding, and micro-cutting, effectively removing material from the surface. As a result, the workpiece surface is treated uniformly and smoothly, achieving a high-quality polishing effect [15].

The MAF (Magnetically Assisted Finishing) technology offers numerous advantages over traditional machining methods, which are particularly evident when handling complex shapes and workpieces with narrow internal walls. It not only enhances processing efficiency but also effectively controls surface quality during machining, achieving precision requirements at the micro and even nano levels [16]. Many scholars, both domestic and international, have conducted extensive research on MAF technology. Misrad [17] combined ultrasonic vibration with a magnetic abrasive finishing process, which significantly improved the surface finishing efficiency of workpieces. Misrad identified two distinct types of forces present during the machining process: a normal force (i.e., indentation force) and a tangential force (cutting force), which directly affect the surface roughness and material removal rate of a workpiece. Through finite element simulations, Misrad analyzed an electromagnet to calculate the magnetic flux density in a working area and assessed the normal force on a workpiece’s surface. The wear theory, combined with ultrasonic vibration effects, was utilized to compute torque during the finishing process. The study predicted the normal force and torque in ultrasonic-assisted magnetic abrasive finishing, relating these forces to the power supply voltage, working gap, and concentration of abrasive particles in flexible magnetic brushes. Ultimately, Misrad validated the accuracy of the theoretical model by comparing it with experimental results.

XinJian Zhang et al. [18] developed a uniquely designed core-shell structure magnetic abrasive particle (MAP) that features an enhanced magnetic saturation rate and improved morphology. By determining the number of active abrasive particles within the MAP, they established a roughness model based on indentation theory. They elaborated on the evolution of the MAF (Magnetically Assisted Forming) mechanism during the elastic–plastic deformation of processed materials, aiming for high efficiency and precision in the MAF processing of slender tube surfaces. XinJian Zhang noted that the grooves on the magnetic pole drive the maximum magnetic force on the MAP. Their theoretical model of magnetic force and the number of active abrasive particles aligned with experimental parameters, effectively predicting and explaining MAF phenomena and mechanisms. For example, the experiments on zirconium alloy tubes demonstrated that after five MAF treatments using the designed MAP, the surface roughness, Ra, improved by a maximum of 63.38%, achieving a final roughness, Ra, of 0.119 μm.

Kala, Prateek, et al. [19] proposed a mathematical model for the double-disk magnetic abrasive finishing (DDMAF) process, designed to effectively process flat paramagnetic workpieces, which are traditionally considered challenging to machines. In their study, surface roughness was modeled as a function of a workpiece’s material properties and various process parameters, including working gap, number of abrasive meshes, abrasive weight percentage, rotational speed, and feed rate. The process model employed Lorentz force and Ampère’s law to estimate the processing force acting on iron particles. These forces were used to calculate the finishing force transferred to the abrasive particles, achieved through the force balance between iron and abrasive particles. Prateek also accounted for the effects of abrasive particle size distribution and frictional force on the finishing force. To determine changes in surface roughness, the research team developed a MATLAB code encompassing all the aforementioned aspects. Their model has been validated against experimental results and applied to study the influence of various process parameters on machining outcomes. Xing, Baijun, and YanHua Zou [20] introduced a method that combines magnetic abrasive finishing with electrolytic magneto rheological polishing (EMAF) to enhance the efficiency of traditional MAF processes. Given that EMAF involves electrochemical reactions, the machining mechanisms vary for different metals. They explored the feasibility of using composite machining tools for the precision surface finishing of aluminum alloy A5052 through a series of experiments and preliminarily investigated its machining mechanisms. Surface roughness and material removal rate were employed to evaluate the finishing effects and efficiency. The current curve of the EMAF process was utilized for assessing and analyzing its machining process. Through simulation analysis and experimental results, the feasibility of EMAF in precision surface finishing was confirmed. Finally, a series of exploratory experiments and parameter optimizations led to the following conclusions: (1) Under identical composite tool and experimental conditions (excluding electrolytic conditions), the EMAF process incorporating electrochemical reactions significantly enhances the finishing efficiency of aluminum alloy A5052 compared to traditional MAF methods. (2) With a working gap set at 1 mm and a NaNO_3_ solution concentration of 15%, the recommended processing voltage is approximately 3.4 V. These findings underscore the effectiveness of the EMAF process in improving precision surface finishing efficiency, supported by both a theoretical analysis and experimental validation.

To solve the difficult problem of traditional processing methods struggling to finish the inner wall of ultra-slender vascular stent tubes, in this study, the plasma melting powder spraying method was used to prepare metal-based Al_2_O_3_ magnetic abrasives with excellent performance, and the magnetic finishing equipment for the inner wall of vascular stent tubes was designed and built. Through a response surface test with four factors and three levels, designed based on the response surface method (Box–Behnken), the influence of tube rotation speed, magnetic pole feed speed, abrasive filling amount, and machining clearance on the surface roughness and their interactions were analyzed. The process parameters were optimized by using the established model and multiple regression equations, and the optimal parameter combination was obtained. This study aims to reduce inner wall surface roughness and eliminate defects, thereby improving surface quality and preparing for the subsequent processes of bare-metal vascular stents.

## 2. Preparation of Plasma Melting Spray Magnetic Abrasives

### 2.1. Principle and Equipment for Magnetic Abrasive Preparation

**Figure 1:**
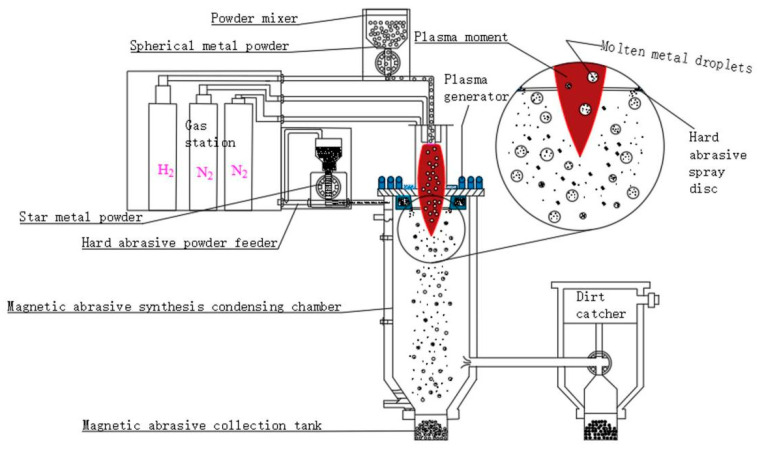
Preparation principle of magnetic abrasives for plasma melt spraying.

Figure 1 illustrates the preparation principle of plasma melting spray magnetic abrasives. The equipment used for this process primarily consists of a plasma generation system, a powder feeding system, a synthesis and collection system, and a gas supply system. Initially, a spherical iron powder that meets specific particle size requirements is selected from raw materials and transported to the plasma generation system via working gas. During this process, the iron powder moves downward through the high-temperature zone of the plasma torch, where it rapidly melts and forms spherical micro-droplets due to surface tension. As the molten iron micro-droplets pass through a spray disk located beneath the plasma generator, they encounter a high-velocity gas stream containing hard abrasive particles. These hard abrasives are propelled onto the surface of the molten micro-droplets, causing them to cool rapidly upon contact and transferring a portion of their thermal energy to the surrounding gas. Ultimately, the molten metal micro-droplets, now infused with hard abrasives, undergo rapid condensation, resulting in high-performance magnetic abrasives characterized by a narrow particle size distribution and high sphericity.

The plasma spraying method was utilized to produce magnetic abrasives, achieving a robust integration between the iron matrix and the hard abrasive particles, resulting in the creation of high-performance spherical magnetic abrasives. This approach combines the advantages of both the atomization method and plasma spraying while incorporating enhancements. The selected spherical iron powder was used as the matrix, ensuring that the magnetic abrasives produced fell within the desired particle size range and mitigating the issue of resource wastage that could arise from insufficient particle sizes in atomization processing. During the preparation process, only the metal matrix was subjected to the high temperatures of the plasma torch, while the hard abrasive particles remained unaffected or were exposed for only a brief period. This preservation of sharpness and the integrity of the hard abrasives prevents the loss of cutting edge due to high temperatures, addressing a common drawback associated with such materials.

### 2.2. Preparation of Iron-Based Aluminum Oxide Magnetic Abrasives

**Figure 2:**
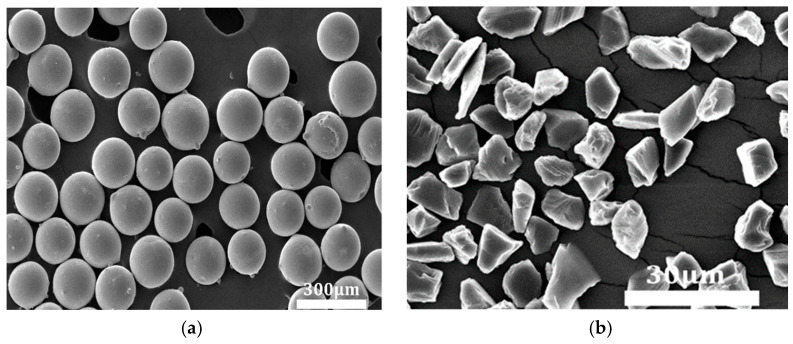
SEM images of spherical iron powder and Al_2_O_3_ powder. (**a**) Metal matrix; (**b**) Al_2_O_3_ hard abrasive powder.

Outstanding magnetic permeability and superior grinding performance are critical for the fabrication of high-performance magnetic abrasives. To ensure process stability during machining, ferromagnetic spherical powder, as illustrated in Figure 2a, was selected as the metallic matrix material. Additionally, the choice of hard abrasives plays a crucial role in the overall effectiveness of the grinding process. As shown in Figure 2b, aluminum oxide (Al_2_O_3_) is widely utilized across various fields due to its exceptional properties, including high hardness, low density, excellent thermal stability, and corrosion resistance [21,22].

### 2.3. Preparation Process

The procedure for fabricating iron-based aluminum oxide magnetic abrasives is as follows: First, an appropriate amount of aluminum oxide powder and iron matrix powder are separately loaded into a hard abrasive feeder and a metal powder feeder. Subsequently, the gas supply valves for both the working gas and powder feed gas are opened, and the power switch is activated. The gas flow rates, working current, and voltage are adjusted as necessary to ensure the stable operation of the plasma torch, while appropriate powder feed pressure is established to initiate the preparation of the magnetic abrasives. Once the fabrication process is complete, the gas supply valves and power switch are turned off, and the powder is collected from the magnetic abrasive collection tank. The sieved magnetic abrasives must be stored in a sealed, dry environment, and the recovered hard abrasive powder should be properly preserved for future use. Throughout the entire process, the selected process parameters listed in Table 1 were employed for the preparation of iron-based aluminum oxide magnetic abrasives.

**Figure 3:**
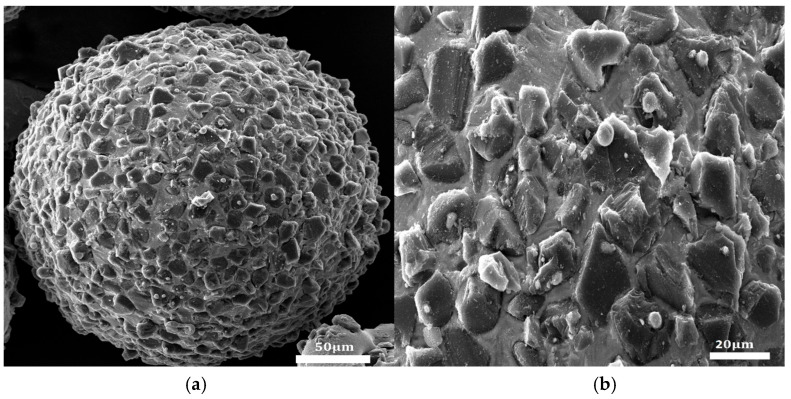
SEM images of iron-based alumina magnetic abrasives. (**a**) Global graph; (**b**) local enlarged graph.

The SEM image of the iron-based alumina magnetic abrasive prepared by the plasma spraying method is shown in Figure 3.

It is evident from the observations that the iron-based aluminum oxide magnetic abrasives produced using this method exhibit an ideal spherical morphology, with the iron matrix’s outer layer densely embedded with uniformly distributed Al_2_O_3_ hard particles. The enlarged images reveal a strong bond between the Al_2_O_3_ hard particles and the iron matrix, with the exposed regions maintaining sharp cutting edges.

## 3. Experiment

### 3.1. Principle of Magnetic Finishing on Inner Wall of Pipe

**Figure 4:**
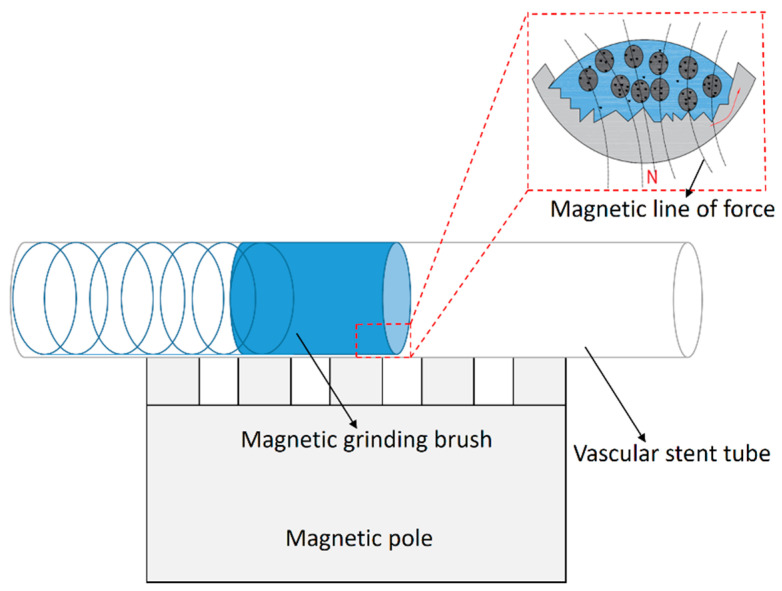
Schematic diagram of magnetic finishing of the inner wall of vascular stent pipe.

Figure 4 illustrates the principle of magnetic polishing processing for the inner wall of vascular stent tubes. Initially, a suitable amount of magnetic abrasive is injected into the interior of the MP35N cobalt–chromium alloy vascular stent tube, which is then sealed at both ends and securely clamped using precision chucks mounted on a servo motor. Under the influence of the magnetic field, the magnetic abrasives within the tube align along the magnetic field lines, forming a flexible magnetic polishing brush. Given that the hardness of the hard particles on the magnetic abrasives is typically much greater than that of the workpiece material, when the magnetic field is applied, these hard particles penetrate the surface of the workpiece, creating indentations and a pressing effect. Subsequently, the tube begins to rotate, while the magnetic poles move back and forth along the tube’s axis, resulting in a helical motion trajectory of the magnetic polishing brush relative to the inner wall. The contact between the hard particles and the workpiece surface generates sliding, cutting, and plowing actions, thereby achieving micro-machining of the tube’s inner wall and facilitating precise material removal.

### 3.2. Mechanism of Magnetic Finishing on the Inner Wall of the Pipe

#### 3.2.1. Dynamic Analysis of Magnetic Abrasives

**Figure 5:**
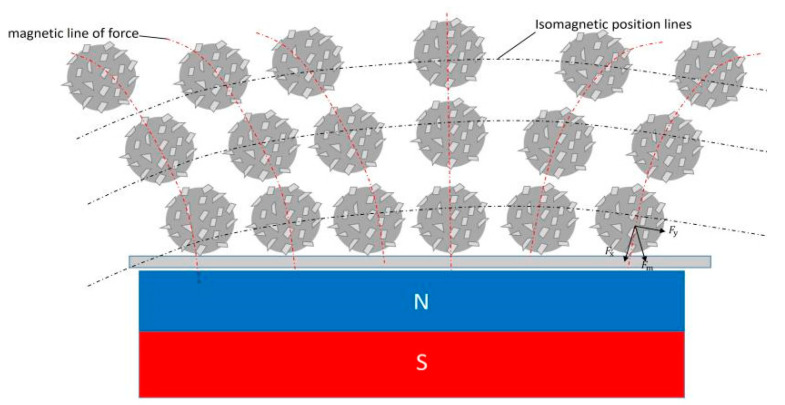
Arrangement and force condition of magnetic abrasive brush when not having started processing.

As illustrated in Figure 5, the magnetic abrasive particles within the tube align along the magnetic flux lines under magnetic field forces, forming a flexible magnetic abrasive brush. Each magnetic abrasive particle experiences dual electromagnetic components: tangential force $F_{x}$ along the magnetic flux lines and normal force $F_{y}$ perpendicular to the magnetic equipotential lines. The resultant magnetic force $F_{m}$, calculated through the vector superposition of these components, initially manifests as a vertically downward orientation perpendicular to the target surface before machining initiation. This pre-machining configuration induces localized plastic deformation through surface indentation, as documented in previous studies [23,24].

**Figure 6:**
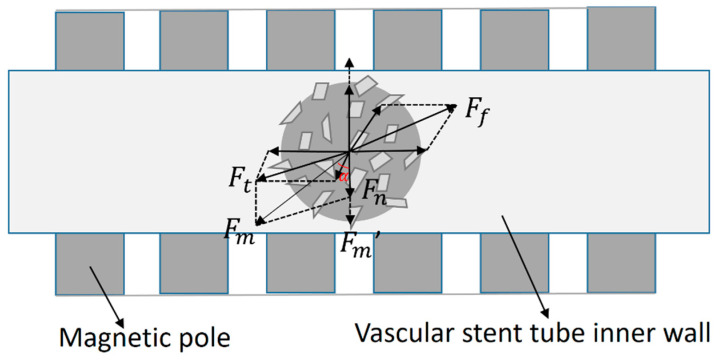
The force of magnetic abrasive powder in the MAF process.

At the start of processing, the tube rotates while the magnetic pole feeds in one direction. The magnetic abrasive experiences a frictional force $F_{f}$ generated by the cutting contact. Simultaneously, the magnitude and direction of the magnetic force $F_{m}$ change due to variations in the magnetic field gradient. The horizontal component of $F_{m}$ becomes equal in magnitude but opposite in direction to the frictional force. As shown in Figure 6, the magnetic force $F_{m}$ forms an angle *α* with a vertical direction. Its horizontal component manifests as the cutting force $F_{t}$, while the vertical component represents the grinding pressure $F_{n}$. The calculation formula is given in Equation (1).

(1) $$\left\{\begin{array}{l} F_{t}=F_{m}\sin\alpha \\ F_{n}=F_{m}\cos\alpha \end{array}\right.$$

The machining boundary condition for material removal is that the cutting force $F_{t}$ exceeds the critical value $F_{r}$ required for material removal, as shown in Equation (2).

(2) $$F_{t}>F_{r}$$

The critical value $F_{r}$ for material removal can be calculated using Formula (3).

(3) $$F_{r}=\frac{\sigma_{y\cdot A_{c}}}{\mu }$$

In the formula, $\sigma_{y}$ is the yield strength of the workpiece; $A_{c}$ is the effective contact area of a single abrasive grain; and μ is the friction coefficient between the abrasive and workpiece.

The critical Angle $\alpha_{r}$ can be calculated using Equation (4).

(4) $$\alpha_{r}=arcsin\frac{F_{r}}{F_{m}}$$

During the initial machining phase, the actuation of magnetic poles precedes abrasive motion while magnetic abrasives maintain quasi-static equilibrium due to frictional constraints $F_{f}$. Concurrently, the progressive angular displacement (*α*) of the resultant magnetic force vector $F_{m}$ relative to the vertical axis emerges from magnetic field gradient evolution. In pre-critical angular configurations ($\alpha <\alpha_{r}$), the tangential cutting force component $F_{t}$ remains sub-critical $\left(F_{t}<F_{r}\right)$, which is insufficient to overcome material cohesion thresholds for micro-scale removal. Upon reaching the critical angular configuration $\left(\alpha \geq \alpha_{r}\right)$, the amplified $F_{t}$ component surpasses the material-specific critical value $F_{r}$, triggering controlled nanoscale material detachment through localized shear plane activation. This phase transition enables magnetic abrasives to overcome static friction limitations, exhibiting hysteretic motion following magnetic pole trajectories with characteristic time-delay parameters, as governed by magneto-rheological principles.

#### 3.2.2. Static Analysis of Magnetic Abrasives

In the grinding area, the calculation formula of the magnetic pressure *P* generated by the magnetic field on the magnetic abrasives is shown in Equation (5).

(5) $$P=\frac{B^{2}}{2\mu_{0}}(1-\frac{1}{\mu_{m}})$$

Among them, *µ_m_* is the relative permeability of the magnetic abrasive, which is composed of the iron-based phase, the hard abrasive particle phase, and air. It can be calculated according to Eucken—a’s electromagnetic theory—as shown in Formula (6).

(6) $$\mu_{m}=\frac{\mu_{a}}{\mu_{0}}\cdot \frac{1-2\left(\delta_{h}\frac{\mu_{a}-\mu_{h}}{2\mu_{a}+\mu_{h}}+\delta_{f}\frac{\mu_{a}-\mu_{f}}{2\mu_{a}+\mu_{f}}\right)}{1+\left(\delta_{h}\frac{\mu_{a}-\mu_{h}}{2\mu_{a}+\mu_{h}}+\delta_{f}\frac{\mu_{a}-\mu_{f}}{2\mu_{a}+\mu_{f}}\right)}$$

In the formula, *µ_a_*, *µ_g_*, *µ_h_* are the magnetic permeabilities (H·m^−1^) of air, hard abrasive grains, and the iron matrix, respectively; *δ_h_* is the volume fraction of the hard abrasive grain phase; and *δ_f_* is the volume fraction of the iron matrix.

Since the magnetic susceptibilities of hard abrasive grains and air are both approximately equal to 0, *µ_a_* = *µ_g_* = *µ_h_* can be simplified. Among them, the relative magnetic permeability of the iron matrix is *µ_r_* = *µ_f_*/*µ*_0_, so Equation (7) can then be obtained.

(7) $$\mu_{m}=\frac{2+\mu_{r}-2\delta_{f}\left(1-\mu_{r}\right)}{2+\mu_{r}+\delta_{f}\left(1-\mu_{r}\right)}$$

Generally speaking, the shapes of magnetic abrasives are not all regular, and the volume fraction of the iron matrix in each magnetic abrasive is also different. For the sake of simplification, generally, the mass fraction of the iron matrix in the magnetic abrasive is multiplied by a coefficient, and this coefficient is generally selected from 0.524 to 0.741. Here, the coefficient is selected as *π*/6, that is, the new volume fraction of the iron matrix is calculated as *πδ_f_*/6. Then, the magnetic pressure on the magnetic abrasive can be finally calculated as follows:

(8) $$P_{n}=\frac{B^{2}}{4\mu_{0}}\cdot \frac{3\pi \left(\mu_{r}-1\right)\cdot \delta_{f}}{3\left(2+\mu_{r}\right)+\pi \left(\mu_{r}-1\right)\cdot \delta_{f}}$$

Use the mass fractions of the iron matrix and hard abrasive grains in the magnetic abrasive to calculate the volume fraction *δ_f_* of the iron matrix.

(9) $$\delta_{f}=\frac{V_{f}}{V_{m}}=\frac{(1-c)\rho_{m}}{\rho_{f}}$$

In the formula, *V_f_* and *V_m_* are the volumes (mm^3^) of the iron matrix and the magnetic abrasive, respectively; *ρ_f_* and *ρ_m_* are the densities (g·cm^−3^) of the iron matrix and the magnetic abrasive, respectively; and *c* is the mass fraction of the hard abrasive grains.

The calculation formulas for the density of the magnetic abrasive and the mass fraction of the hard abrasive grains are shown in Equation (10).

(10) $$\left\{\begin{array}{l} \rho_{m}=\frac{\rho_{f}\cdot \rho_{h}}{c\cdot \rho_{f}+(1-c)\cdot \rho_{h}}\\ c=\frac{W_{h}}{W_{h}+W_{f}} \end{array}\right.$$

In the formula, *W_h_* and *W_f_* are the masses (g) of the hard abrasive grains and the iron matrix, respectively, and *ρ_h_* is the density (g·cm^−3^) of the hard abrasive grains.

#### 3.2.3. Material Removal Mechanism Analysis

**Figure 7:**
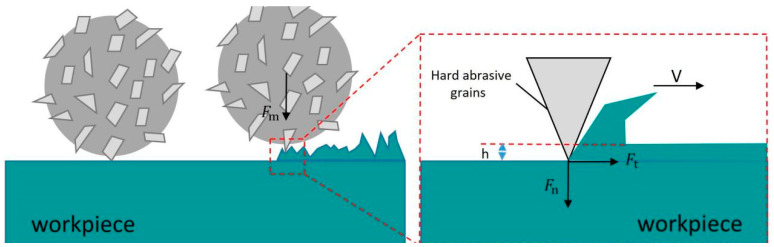
Micro-cutting extrusion model of single abrasive grain.

The material removal mechanism in magnetic abrasive finishing (MAF) of tube inner walls primarily originates from coordinated micro-scale material removal through tribological interactions between magnetic abrasives and the workpiece surface, as schematically depicted in Figure 7. Under magnetically induced compressive stress, the abrasive particles exert dual mechanical actions: (1) normal force component $F_{n}$, which generates localized plastic deformation through indentation depth *h*, and (2) tangential force component $F_{t}$, which is responsible for controlled abrasive progression along the machining path. When geometrically optimized abrasive grains achieve proper spatial orientation, their protrusions function as micro-cutting tools, inducing precision material removal at submicron scales.

While individual abrasive particles exhibit stochastic and intermittent cutting behavior, the magnetic abrasive brush demonstrates remarkable self-regenerative characteristics. Through synergistic interactions between magnetic field dynamics and rotational kinematics, the abrasives undergo continuous spatial reconfiguration via three-dimensional particle rotation, positional realignment, and cutting-edge renewal. This autonomous reorganization ensures the persistent engagement of active cutting edges, thereby sustaining stable material removal rates while mitigating surface integrity degradation.

The material removal mechanism and its computational modeling in the magnetic abrasive finishing (MAF) of tube inner walls present significant complexity due to multi-physics interactions. To derive an analytical solution for indentation depth *h*, fundamental assumptions are adopted in establishing the numerical model:

1. Both magnetic abrasives and hard abrasive grains exhibit ideal spherical geometry;
2. Hard grains are partially embedded (50% volumetric ratio) within the ferromagnetic matrix with uniform dispersion and topological arrangement;
3. Continuous micro-cutting conditions prevail during processing.

**Figure 8:**
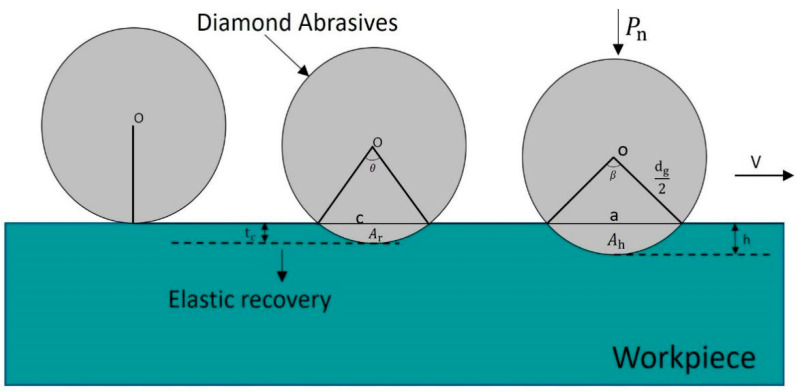
Contact model between hard abrasive particles and workpiece surface.

As illustrated in Figure 8, the abrasive–workpiece contact mechanics follows an elastic–plastic framework.

The calculation of the normal force $F_{n}$ exerted by a single abrasive grain on the workpiece surface is presented in Equation (11).

(11) $$F_{n}=P_{n}\cdot \frac{\pi d_{g}^{2}}{4}$$

In the formula, *d_g_* represents the diameter (mm) of the hard abrasive grains.

The flow stress *σ_w_* of the workpiece material can be calculated from the Brinell hardness *H_B_* of the material:

(12) $$\sigma_{w}=K\cdot H_{B}$$

In the formula, *K* is a coefficient determined by the material type [25].

It can be seen from Figure 8 that the calculation formula for the indentation area where a single hard abrasive grain contacts the workpiece surface is as shown in Equation (13).

(13) $$A_{p}=\frac{\pi a^{2}}{4}=\pi \left(d_{g}h-h^{2}\right)$$

In the formula, *a* represents the chord length (mm) corresponding to the indentation depth.

According to Equations (11)–(13), the normal force can be calculated as follows:

(14) $$F_{n}=\sigma_{w}\cdot A_{p}=RH_{B}\pi \left(d_{g}h-h^{2}\right)$$

From the above equations, the indentation depth h can be calculated using Equation (15).

(15) $$h=\frac{d_{g}}{2}-\sqrt{\frac{d_{g}^{2}}{4}-\frac{P_{n}d_{g}^{2}}{4RH_{B}}}$$

During the magnetic finishing process, there is always a certain amount of elastic deformation in the contact area between the hard abrasive grains and the workpiece surface, as shown in Figure 8, which can be calculated according to the Hertz contact theory (16).

(16) $$t_{c}={\left(\frac{9}{16}\cdot \frac{1}{d_{g}/2}\right)}^{1/3}\cdot {\left(\frac{P_{n}}{E_{r}}\right)}^{2/3}$$

In the formula, $E_{r}$ is the elastic modulus of the contact area, and its calculation formula is shown in Equation (17).

(17) $$\frac{1}{E_{r}}=\frac{1-v_{k}^{2}}{E_{k}}+\frac{1-v_{i}^{2}}{E_{i}}$$

In the formula, *v_k_* and *v_i_* are the Poisson’s ratios of the workpiece and the hard abrasive grains, respectively, and *E_k_* and *E_i_* are the elastic moduli of the workpiece and the hard abrasive grains, respectively.

The calculation formulas for the indentation area and elastic recovery area removed by a single abrasive grain are shown in Equation (18).

(18) $$\left\{\begin{array}{c} A_{h}=\frac{d_{g}^{2}}{8}(\beta -\sin\beta )\\ A_{t}=\frac{d_{g}^{2}}{8}(\theta -\sin\theta ) \end{array}\right.$$

In the formula, *β* and *θ* are the central angles corresponding to the indentation area and the elastic recovery area, respectively. They can also be calculated through the chord length as follows using Equation (19):

(19) $$\left\{\begin{array}{c} A_{h}=\frac{2}{3}ah+\frac{h^{3}}{2a}\\ A_{t}=\frac{2}{3}ct_{c}+\frac{t_{c}^{3}}{2c} \end{array}\right.$$

In the formula, *a* and *c* are the chord lengths corresponding to the indentation area and the elastic recovery area, respectively. The calculation formula is shown in Equation (20).

(20) $$\left\{\begin{array}{c} a=2\sqrt{h\left(d_{g}-h\right)}\\ c=2\sqrt{t_{c}\left(d_{g}-t_{c}\right)} \end{array}\right.$$

Therefore, the total material removal area of a single magnetic abrasive grain can be calculated as follows using Equation (21):

(21) $$A_{r}=A_{h}+A_{c}$$

### 3.3. Equipment and Materials

#### 3.3.1. Equipment

**Figure 9:**
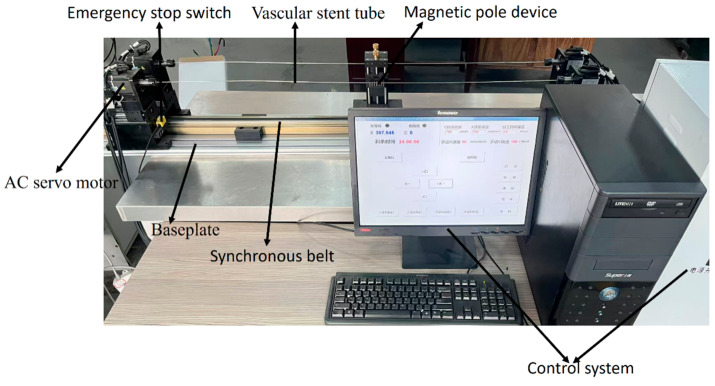
Cobalt–chromium alloy vascular stent tube inner wall magnetic finishing device.

Figure 9 presents the magnetic polishing apparatus developed for the inner wall of cobalt–chromium alloy vascular stent tubes. Figure 6 illustrates the control system for the magnetic abrasive polishing of the inner surface of these stent tubes. This equipment comprises four main components: the tube rotation mechanism, the magnetic pole assembly, the magnetic pole feed system, and the control system. Firstly, the tube rotation mechanism employs a precision chuck with a machine shaft to securely grip the stent tube, which is tightened using a tensioning device. This mechanism is powered by an AC servo motor, ensuring precise axial feeding and the rotation of the tube workpiece relative to the magnetic poles. Secondly, the magnetic pole assembly utilizes N35 neodymium–iron–boron magnets, which effectively drive the magnetic abrasives in an axial reciprocating motion at a predetermined feed rate. The movement of the magnetic poles is controlled through a linear module with a synchronous belt, stepper motor, and proximity switch, ensuring a long stroke and excellent load-bearing capacity. Lastly, the control system is built on an open CNC software architecture developed on the Windows operating system platform, optimized using VC++ and Delphi programming languages for virtual device drivers and Windows threads. This design ensures that the control system meets the stringent requirements of the magnetic polishing apparatus for the inner walls of vascular stent tubes. The integration of these components enables the device to efficiently perform magnetic polishing, enhancing both processing quality and efficiency.

#### 3.3.2. Materials

**Figure 10:**
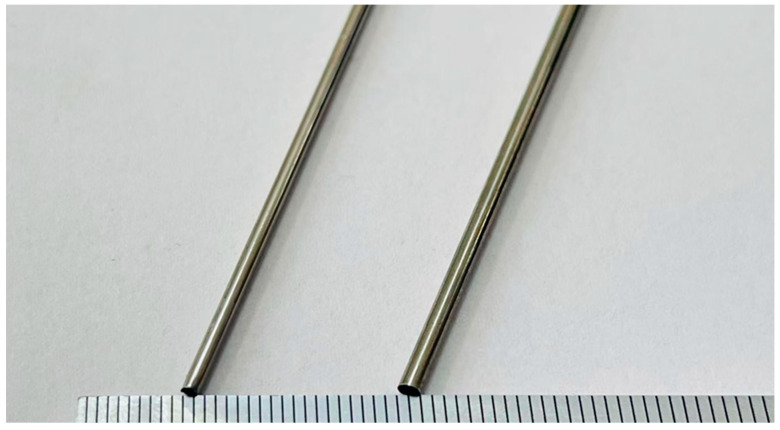
Test MP35N cobalt–chromium alloy vascular stent pipe physical picture.

The MP35N cobalt–chromium alloy vascular stent tube used in the experiment is depicted in Figure 10. Its outer diameter is 1.8 mm, inner diameter is 1.6 mm, and length ranges from 1800 mm to 2000 mm. The composition and certain performance specifications of the alloy are detailed in Table 2 and Table 3. It is particularly noteworthy that the MP35N cobalt–chromium alloy is non-ferromagnetic, thus eliminating concerns regarding interference between the magnetic poles and the workpiece.

### 3.4. BBD Experimental Design

The Response Surface Methodology (RSM), proposed by Box et al., is a statistical technique for experimental design that integrates experimental design with mathematical modeling to optimize processes [26]. This approach involves conducting experiments at representative local points to regression-fit the functional relationship between factors and outcomes over the entire range, ultimately identifying the optimal levels of each factor [27]. For this study, a Box–Behnken Design (BBD) was employed to establish and analyze the response surface. The Box–Behnken design is a three-factor experimental design that involves fewer repetitions of the central points, facilitating the estimation of experimental error and the identification of quadratic terms in the model, making it suitable for response surface optimization [28,29]. Using Design Expert 13 software, a BBD experiment was designed with four factors at three levels. Table 4 outlines the factor levels and corresponding coded values for the BBD experimental parameters.

### 3.5. Test Results

The surface roughness value of the inner wall of the processed MP35N cobalt–chromium alloy stent is the response value, and the test results are shown in Table 5.

The experimental data were fitted using Design Expert 13 software to obtain the variance analysis results for the regression prediction model of surface roughness on the inner wall of the processed MP35N cobalt–chromium alloy tubing, as shown in Table 6. A “Prob > F” value, or *p*-value, less than 0.05 indicates that the corresponding source of variance significantly affects the established regression equation. When the *p*-value is below 0.0001, the model is highly significant; conversely, a *p*-value greater than 0.05 suggests that the model is not significant.

From the variance analysis results presented in Table 6, it can be observed that, in terms of individual parameters, the model’s F-value is 32.07 with a *p*-value less than 0.0001, indicating a strong fit between the surface roughness of the model and the independent variables included in the regression equation. The lack of fit F-value is 1.18, and the corresponding *p*-value is 0.5426 (greater than 0.05), demonstrating that the lack of fit is not significant when compared to pure error. The coefficient of determination (R^2^) is 0.9674, and the adjusted R^2^ value is 0.9554, suggesting that this regression model can explain 95.54% of the response values, indicating a high degree of fit. Therefore, the roughness values predicted by this model for the inner surface of the processed tubing have a high level of reliability, affirming the validity of the model.

According to the data presented in Table 6, the *p*-values for the tubing rotation speed (A), magnetic pole feed rate (B), magnetic abrasive filling amount (C), and machining gap (D) are 0.0021, 0.0527, 0.0046, and 0.0008, respectively. This indicates that the influence of these four machining parameters on surface roughness follows the following order: D > A > C > B. Since the *p*-values for all four process parameters are less than 0.05, it can be concluded that their effects on surface roughness are significant. The multiple regression equation correlating surface roughness with tubing rotation speed (A), magnetic pole feed rate (B), magnetic abrasive filling amount (C), and machining gap (D) is given in Equation (22).

(22) $$\begin{array}{ll} Ra= & 0.099-6.667\times 10^{-3}A-3.667\times 10^{-3}B-5.917\times 10^{-3}C+3.417\times 10^{-3}D+2.500\times 10^{-4}AB\\ & -3.500\times 10^{-3}AC+1.250\times 10^{-3}AD+2.000\times 10^{-3}BC-2.500\times 10^{-4}BD\\ & +2.250\times 10^{-3}CD+0.050A^{2}+0.010B^{2}+0.019C^{2}+0.037D^{2} \end{array}$$

**Figure 11:**
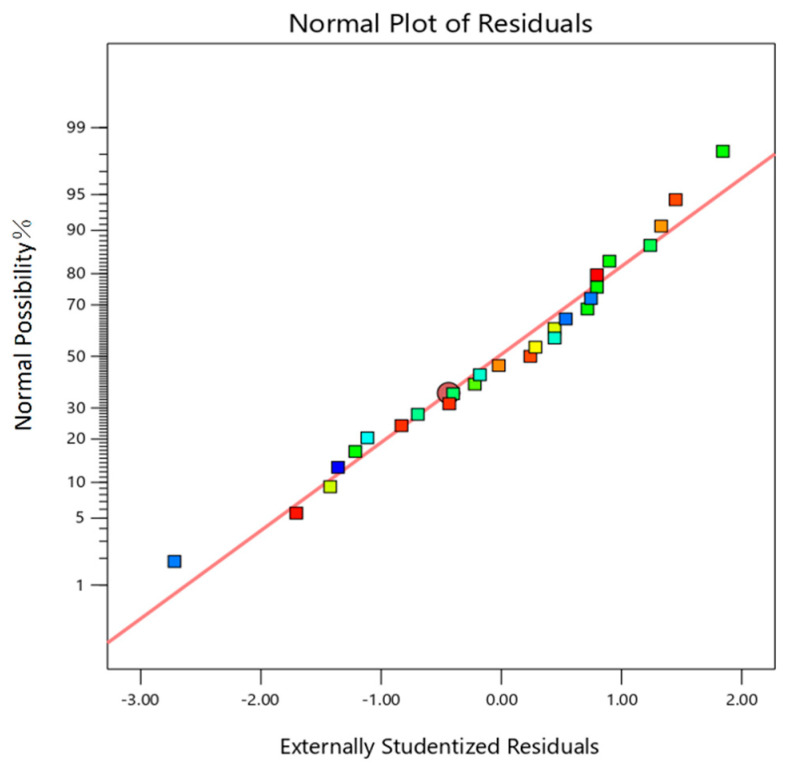
Residual normal distribution diagram.

As illustrated in Figure 11, the residuals of the model exhibit a nearly linear pattern in terms of normal distribution. This indicates a strong alignment between the predicted and actual values of surface roughness, suggesting that the residuals follow a normal distribution and that the model demonstrates good adaptability.

### 3.6. Analysis of Experimental Results

A second-order response surface mathematical model was developed using factors such as tubing rotation speed, magnetic pole feed rate, magnetic abrasive filling amount, and machining gap. By fixing two of these factors, the interactive effects of the remaining variables on surface roughness were systematically investigated. The interaction effects among the factors were evaluated through the analysis of contour shapes and curve densities.

**Figure 12:**
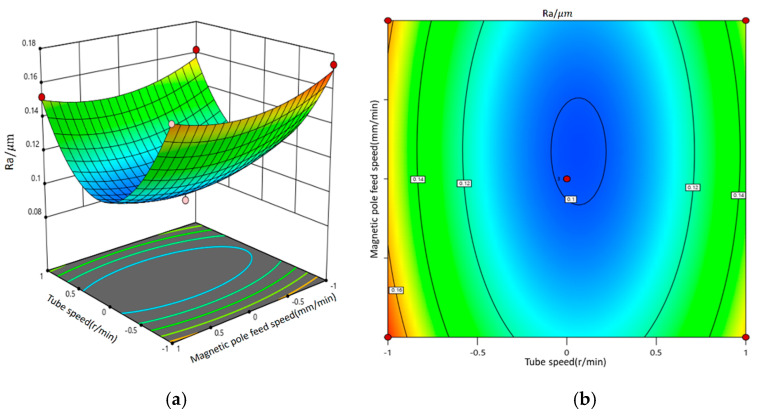
The interaction diagram of pipe speed and magnetic pole feed speed and its impact on surface roughness. (**a**) Three-dimensional response surface diagram; (**b**) contour map.

Figure 12 presents the three-dimensional response surface and contour plot illustrating the interaction between tubing rotation speed and magnetic pole feed rate and its impact on surface roughness. The findings reveal that when both the magnetic abrasive filling amount and machining gap are maintained at mid-range values, neither excessively low nor high tubing rotation speeds or magnetic pole feed rates can achieve optimal surface roughness. Notably, the impact of tubing rotation speed on surface roughness is significantly greater than that of the magnetic pole feed rate. Adjustments to the tubing rotation speed and magnetic pole feed rate alter the operational trajectory of the magnetic abrasive brush; however, due to the pronounced effect of tubing rotation speed, it is sufficient to maintain the magnetic pole feed rate within a range that does not affect the positioning of the magnetic abrasives to control surface roughness effectively. High tubing rotation speeds may lead to instability in the magnetic abrasive brush, making it challenging to sustain consistent machining efficacy, while low speeds may result in excessive grinding of the inner wall of the tubing, increasing material removal rates. Therefore, selecting the appropriate tubing rotation speeds and magnetic pole feed rates is crucial.

**Figure 13:**
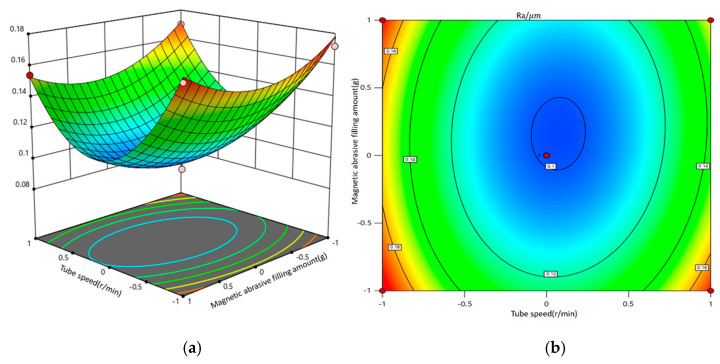
The interaction diagram of pipe speed and magnetic abrasive filling amount and its impact on surface roughness. (**a**) Three-dimensional response surface diagram; (**b**) contour map.

Figure 13 illustrates a three-dimensional response surface and contour plot depicting the impact of the interaction between tubing rotation speed and magnetic abrasive filling amount on surface roughness. When the magnetic pole feed rate and machining gap are set to mid-range values, an optimal combination of tubing rotation speed and magnetic abrasive filling amount significantly enhances surface roughness. Insufficient magnetic abrasives may hinder the effective formation of a robust magnetic abrasive brush, compromising machining efficiency and leading to suboptimal results if maintenance is neglected. Conversely, an excessive amount of magnetic abrasives, constrained by the limited space within the tubing, can cause blockages that disrupt the machining process. Therefore, selecting an appropriate balance of tubing rotation speed and magnetic abrasive filling amount is essential for achieving optimal machining outcomes.

**Figure 14:**
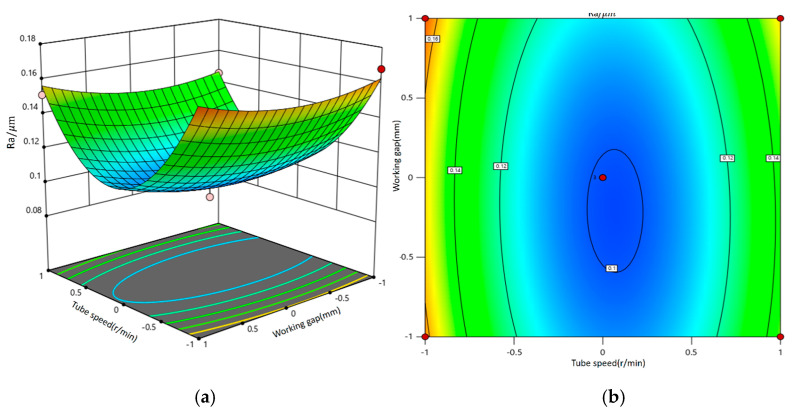
Interaction diagram of tube speed and machining gap and its impact on surface roughness. (**a**) Three-dimensional response surface diagram; (**b**) contour map.

Figure 14 presents the three-dimensional response surface and contour plot illustrating the impact of the interaction between tubing rotation speed and machining gap on surface roughness. When the abrasive filling amount and abrasive feed rate are fixed at mid-range values, an increase in tubing rotation speed and machining gap correlates with a rising trend in surface roughness values. Consequently, achieving lower surface roughness is contingent upon selecting appropriate tubing rotation speeds and machining gaps concurrently. The influence of the machining gap on surface quality is more pronounced than that of tubing rotation speed, as a smaller machining gap enhances magnetic induction strength, thereby increasing the force on the magnetic abrasives and effectively improving material removal efficiency.

### 3.7. Optimization and Validation

To achieve the objective of minimizing the surface roughness of the inner wall of the tubing after magnetic finishing, data simulation and optimization were performed on the experimental results. The mathematical model predicts a minimum surface roughness value of Ra 0.104 µm. The optimized process parameters are as follows: a tubing rotation speed of 600 r/min, a magnetic pole feed rate of 150 mm/min, a magnetic abrasive filling amount of 0.150 g, and a machining gap of 0.500 mm. To validate the accuracy of the response surface methodology in predicting machining parameters, verification experiments were conducted using the rounded process parameters, with specific results shown in Table 7.

**Figure 15:**
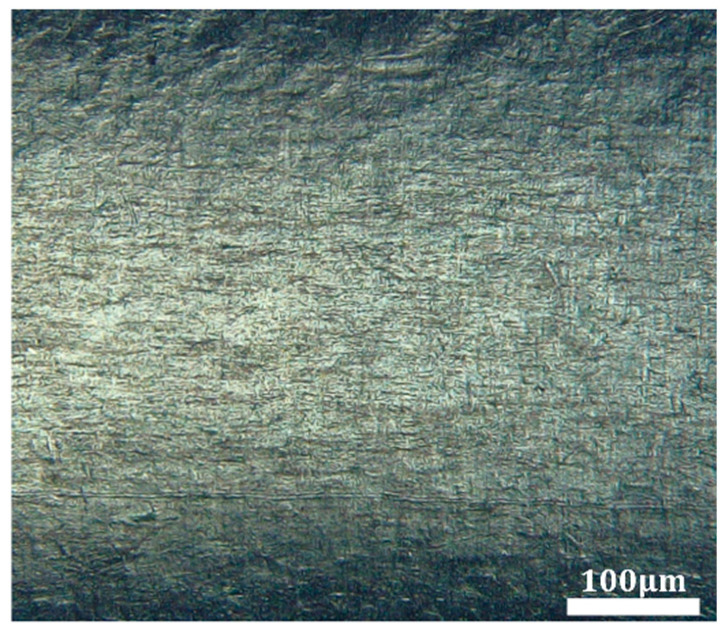
Initial SEM images of the inner wall of an MP35N cobalt–chromium alloy stent tube.

Using a DSX1000 super-depth 3D microscope, both 2D and 3D morphological observations were conducted on the inner surface of the tubing, and the surface roughness was measured. Subsequently, a scanning electron microscope (SEM) was employed for further examination of the inner wall surface. Figure 15 presents the initial SEM images of the MP35N cobalt–chromium alloy vascular stent tubing, clearly revealing defects on the surface, such as protrusions, pits, and scratches, with a surface roughness measurement of 0.486 µm.

**Figure 16:**
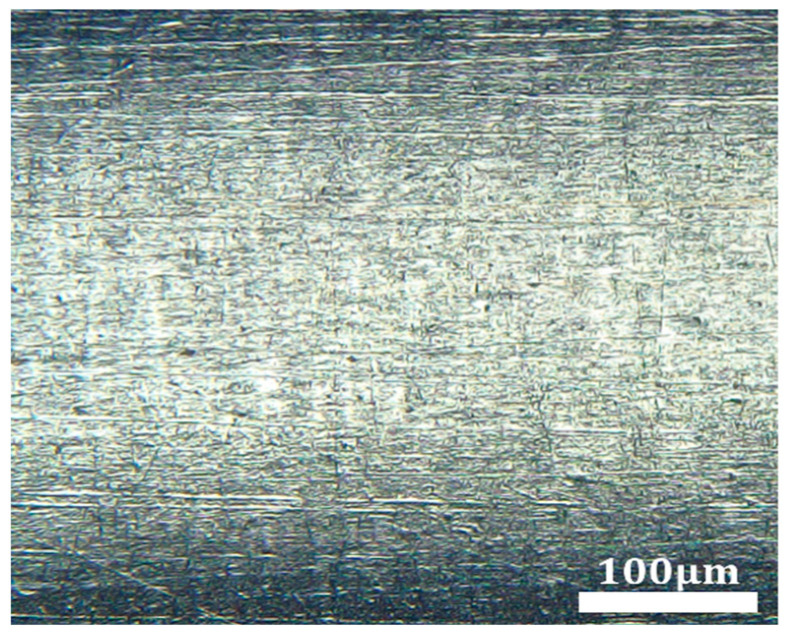
SEM images of the inner wall of MP35N cobalt–chromium alloy vascular stent tubing from the 15# experimental scheme.

Figure 16 presents the SEM images of the inner wall of the tubing obtained from the 15# experimental scheme in the BBD study. As observed, under the optimized process parameters, the magnetic finishing process resulted in a significantly smoother and more even surface on the tubing’s inner wall. The original defects, such as protrusions and pits, have largely disappeared, indicating a substantial improvement in surface quality. However, several scratches remain, with a measured surface roughness value of 0.122 µm.

**Figure 17:**
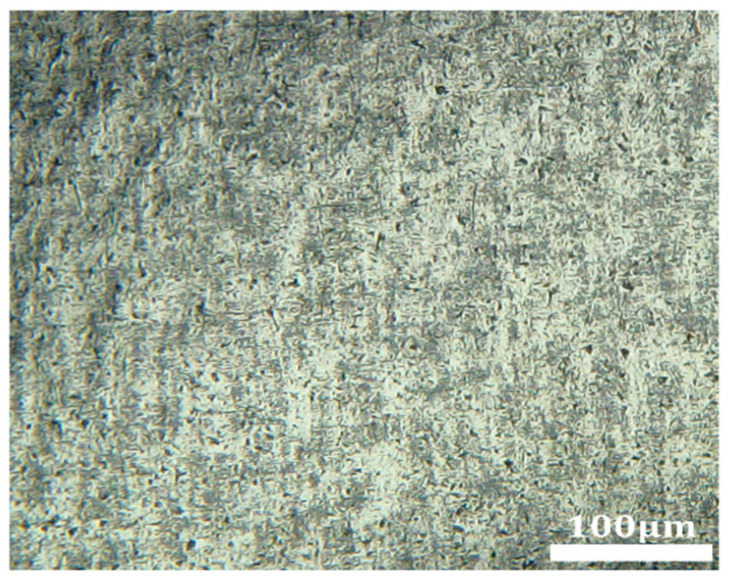
SEM image of the inner wall of an MP35N cobalt–chromium alloy vascular stent tube after optimization.

Figure 17 illustrates the surface morphology after processing with the optimized parameters. Compared to Figure 16, there is a noticeable reduction in the number of scratches, and the surface appears more uniform and smoother. This indicates a further enhancement in surface quality, with a measured roughness value of 0.104 µm.

## 4. Conclusions

Based on this study, the following conclusions can be drawn:

(1) An innovative process was developed for the in situ synthesis of Al_2_O_3_-reinforced iron-based magnetic abrasives via plasma molten spray powder technology. Through precise control of the melting–solidification process, uniform particle size distribution was achieved, resulting in abrasives with high sphericity (>92%) and gradient composite structure. Microstructural characterization confirmed the dense encapsulation of Al_2_O_3_ on the iron matrix surface. This “rigid–soft” coupling characteristic endowed the abrasives with a sustained cutting capability and exceptional impact resistance during processing.
(2) A quaternary quadratic surface roughness prediction model was established based on the Box–Behnken response surface methodology, exhibiting a determination coefficient of R^2^ = 0.9554 and a prediction error of <3%. This study revealed the nonlinear coupling mechanisms of the process parameters in magnetic abrasive finishing (MAF): The machining gap dominated surface quality regulation with a weighting factor of 52.7%, while its interaction with tube rotation speed demonstrated a synergistic enhancement effect on material removal mechanisms. Through multi-objective optimization, a globally optimal parameter combination was obtained, and experimentally verified to reduce the inner surface roughness of MP35N tubes from Ra 0.486 μm to Ra 0.107 μm (78% reduction), with an over 90% elimination rate of surface defect layers.
(3) A dedicated magnetic abrasive finishing device for tube inner walls was developed, which effectively removed the defect layers from MP35N cobalt–chromium alloy vascular stent tubes, significantly reduced surface roughness (Sa < 0.12 μm), and enhanced surface integrity. This study provides critical technical guidance and reference value for manufacturing high-quality vascular stent tubes and other medical catheters.
(4) Future research will focus on exploring complex-shaped tubular component processing and integrating online monitoring technologies to advance magnetic abrasive finishing toward intelligent and flexible developments. Subsequent studies should also address in vivo fatigue performance validation and biocompatibility optimization for clinical translation.

## Figures and Tables

**Table 1:** Iron-based Al_2_O_3_ magnetic abrasive preparation test process parameters.

Process Variable	Parameter Setting
Spray outlet angle (°)	65
Sprinkler ring joint diameter (mm)	3.5
Nozzle aperture (mm)	46
Sprinkler inlet pressure (MPa)	0.8
Distance between spray plate and plasma generator (mm)	65
Current (A)	700
Argon flow rate (L/h)	800
Hydrogen flow rate (L/h)	80
Powder feeding rate of iron ball powder (g/min)	45
Powder feeding rate of Al_2_O_3_ powder (g/min)	270
Equipment power (kW)	24.54

**Table 2:** Composition table of MP35N cobalt–chromium alloy vascular stent tube.

Element	Co	Cr	Mo	Ni	C	Mn	Si	Fe
Mass fraction (%)	34	20	9.75	35	≤0.15	≤0.15	≤0.15	≤1

**Table 3:** Performance indexes of L605 cobalt–chromium alloy vascular stent tube.

Performance Index	Density (g·cm^−3^)	Elastic Modulus (GPa)	Tensile Strength (MPa)	Yield Strength (MPa)	Elongation (%)	Fatigue Limit (GPa)
Numerical value	8.43	207	1200–1860	1170–1580	20–30	600–900

**Table 4:** Correspondence table of factor level and coding value of BBD.

Coded Value	A-Pipe Speed (r/min)	B-Pole Velocity (mm/min)	C-Magnetic Abrasive Filling Amount (g)	D-Machining Gap (mm)
−1	400	50	0.1	0.5
0	600	100	0.15	1
1	800	150	0.2	1.5
Amplitude of variation	200	50	0.05	0.5

**Table 5:** Test plan and results.

Test Serial Number	A-Spindle Speed (r/min)	B-Pole Velocity (mm/min)	C-Magnetic Abrasive Filling Amount (g)	D-Machining Gap (mm)	Surface Roughness Ra (µm)
1	400	50	0.15	1	0.174
2	800	50	0.15	1	0.162
3	400	150	0.15	1	0.163
4	800	150	0.15	1	0.152
5	600	100	0.1	0.5	0.138
6	600	100	0.2	0.5	0.117
7	600	100	0.1	1.5	0.137
8	600	100	0.2	1.5	0.125
9	400	100	0.15	0.5	0.168
10	800	100	0.15	0.5	0.146
11	400	100	0.15	1.5	0.168
12	800	100	0.15	1.5	0.151
13	600	50	0.1	1	0.136
14	600	150	0.1	1	0.132
15	600	50	0.2	1	0.122
16	600	150	0.2	1	0.126
17	400	100	0.1	1	0.172
18	800	100	0.1	1	0.17
19	400	100	0.2	1	0.17
20	800	100	0.2	1	0.154
21	600	50	0.15	0.5	0.114
22	600	150	0.15	0.5	0.103
23	600	50	0.15	1.5	0.129
24	600	150	0.15	1.5	0.117
25	600	100	0.15	1	0.093
26	600	100	0.15	1	0.103
27	600	100	0.15	1	0.102

**Table 6:** ANOVA results of surface roughness.

Source of Variance	Sum of Squares	Degree of Freedom	Mean Square Value	F	Prob > F	Prominence
Model	0.016	14	1.12 × 10^−3^	32.07	<0.0001	Prominence
A-Tube speed	5.333 × 10^−4^	1	5.333 × 10^−4^	15.28	0.0021	
B-Magnetic pole feed rate	1.613 × 10^−4^	1	1.613 × 10^−4^	4.62	0.0427	
C-magnetic abrasive filling amount	4.201 × 10^−4^	1	4.201 × 10^−4^	12.03	0.0046	
D-machining gap	5.741 × 10^−4^	1	5.741 × 10^−4^	26.53	0.0008	
AB	2.5 × 10^−7^	1	2.5 × 10^−7^	0.007161	0.934	
AC	4.9 × 10^−5^	1	4.9 × 10^−5^	1.4	0.2591	
AD	6.25 × 10–_6_	1	6.25 × 10^−6^	0.18	0.6797	
BC	1.6 × 10^−5^	1	1.6 × 10^−5^	0.46	0.5112	
BD	2.5 × 10^−7^	1	2.5 × 10^−7^	0.007161	0.934	
CD	2.025 × 10^−5^	1	2.025 × 10^−5^	0.58	0.461	
A2	1.4 × 10^−2^	1	1.4 × 10^−2^	389.61	<0.0001	
B2	5.88 × 10^−4^	1	5.88 × 10^−4^	16.84	0.0015	
C2	1.9151 × 10^−3^	1	1.9151 × 10^−3^	55.88	<0.0001	
D2	3.741 × 10^−4^	1	3.741 × 10^−4^	10.72	0.0067	
Residuals	4.189 × 10^−4^	12	3.491 × 10^−5^			
Misfit error	3.583 × 10^−4^	10	3.583 × 10^−5^	1.18	0.5426	Non-significant
Pure error	6.067 × 10^−5^	2	3.033 × 10^−5^			
Total	0.016	26				
R^2^ = 0.9674			Adj R^2^ = 0.9554			

**Table 7:** Experimental results after parameter optimization.

Experiment 1	Experiment 2	Experiment 3	Average Value	Predicted Value	Error
0.108 μm	0.102 μm	0.110 μm	0.107 μm	0.104 μm	2.9%

## Data Availability

The datasets used or analyzed during the current study are available from the corresponding author on reasonable request.
